# P-1882. Answering the Call: Volume and Characteristics of External Phone Consultations to Pediatric Infectious Diseases Specialists at a Large Academic Children's Hospital

**DOI:** 10.1093/ofid/ofae631.2043

**Published:** 2025-01-29

**Authors:** Joana Dimo, Erin C Ho, Juri Boguniewicz, Justin B Searns, Kevin Messacar, Jessica R Cataldi

**Affiliations:** University of Colorado/Children's Hospital Colorado, Denver, Colorado; University of Colorado School of Medicine, Aurora, CO; University of Colorado School of Medicine, Aurora, CO; Children’s Hospital Colorado, University of Colorado, Aurora, Colorado; University of Colorado, Children’s Hospital Colorado, Aurora, Colorado; University of Colorado School of Medicine, Aurora, CO

## Abstract

**Background:**

Due to the limited supply and geographic distribution of pediatric infectious diseases (Peds ID) specialists, providing unremunerated telephone advice to outside clinicians has become a significant component of day-to-day practice for many academic Peds ID clinicians. This time-consuming service is not classified as direct patient care or adequately captured by traditional billing and productivity metrics. The objective of this study was to quantify and characterize external phone consults to Peds ID specialists at Children’s Hospital Colorado (CHCO), and compare the burden of these phone consults to other pediatric subspecialists.

**Figure:**
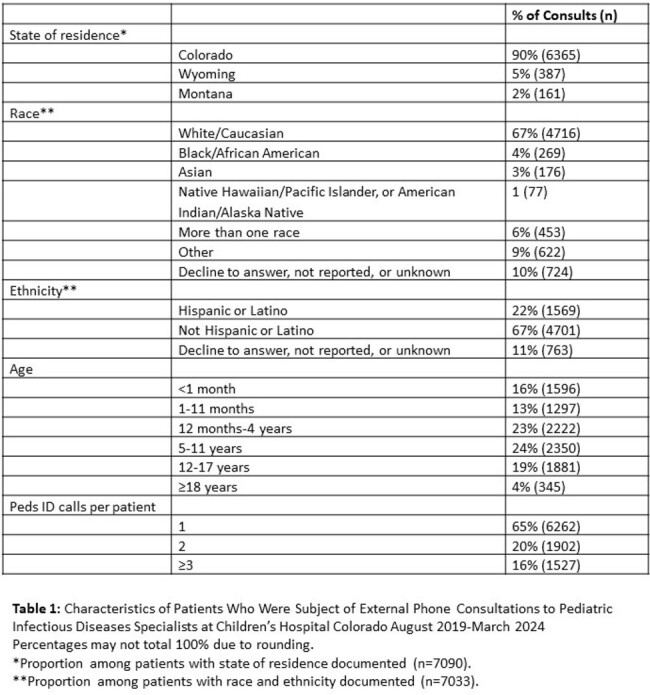

**Methods:**

External phone consults to pediatric subspecialists at CHCO from April 2019-March 2024 were obtained from the electronic medical record (EMR). Patient and call characteristics for Peds ID were summarized with descriptive statistics.

**Figure:**
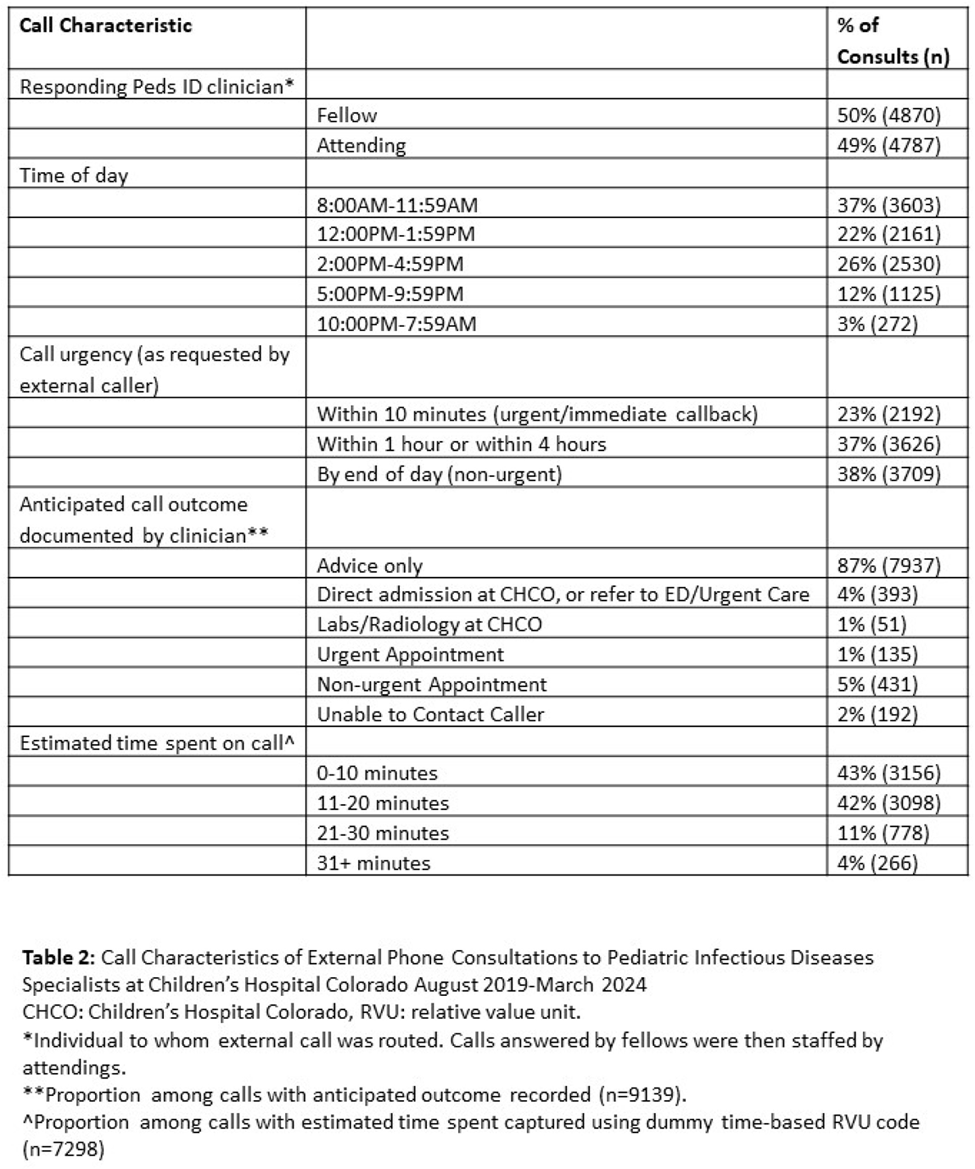

**Results:**

From 2021-2023, Peds ID received the second most external phone consults (n=6376) of any pediatric subspecialty. From 2019 to 2024, average calls to Peds ID increased 21% from 38 to 46 calls/week. One-quarter of calls were about patients registered in the EMR only for the phone encounter and never for direct patient care. Most calls concerned children < 5 years and 9% were for children 0-7 days old. Eighty-five percent of calls occurred between 8AM-5PM and 23% requested immediate callback. Fridays had the most calls (mean 7.6 calls/day), and Sundays had the least (2.1). Based on dummy time-based RVU codes, the median call time was 11-20 minutes; 36% of calls required at least one follow-up call. Only 11% of calls were expected to lead to direct patient care at CHCO based on clinician recommendation. Peds ID specialists spent over 2000 unbilled hours on external phone consults over the study period and an average of > 10 hours/week in the most recent year of data.

**Figure:**
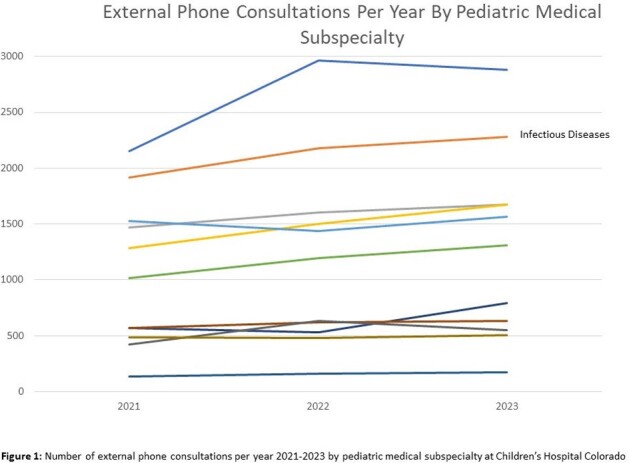

**Conclusion:**

External phone consults to Peds ID at CHCO are increasing and time-consuming, yet do not directly generate revenue and do not reliably lead to direct patient care in our health system. Given limited supply, increased demand, and reimbursement challenges for Peds ID clinicians, different economic models for external phone consults should be explored. Chart review to describe callers, reasons for calls, and patient outcomes may inform these efforts.

**Figure:**
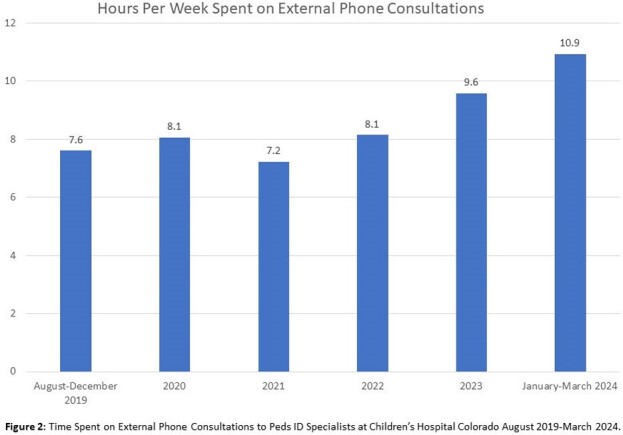

**Disclosures:**

Juri Boguniewicz, MD, AstraZeneca: Grant/Research Support

